# Erratum to: Haloperidol Use Among Elderly Patients Undergoing Surgery: A Retrospective 1-Year Study in a Hospital Population

**DOI:** 10.1007/s40801-016-0069-2

**Published:** 2016-04-11

**Authors:** Harmke Nijboer, Geert Lefeber, Alasdair MacLullich, Barbara van Munster

**Affiliations:** Catharina Hospital, Eindhoven, Brabant The Netherlands

## Erratum to: Drugs - Real World Outcomes (2016) 3:83–88 DOI 10.1007/s40801-016-0060-y

An Online First version of this article was made available online at http://link.springer.com/journal/40801/onlineFirst/page/1 on 3 March 2016. An error was subsequently identified in the article, and the following correction should be noted:

**Page 1, author listing**: The name of the third author, which previously read:

“Alidair McLullich”

should read:

“Alasdair MacLullich”

**Page 5, Conflict of interest**: The name of the first author, which previously read:

“Prof A McLullich”

should read:

“Prof A MacLullich”.

